# ABT-MPNN: an atom-bond transformer-based message-passing neural network for molecular property prediction

**DOI:** 10.1186/s13321-023-00698-9

**Published:** 2023-02-26

**Authors:** Chengyou Liu, Yan Sun, Rebecca Davis, Silvia T. Cardona, Pingzhao Hu

**Affiliations:** 1 Department of Electrical and Computer Engineering, University of Manitoba, Winnipeg, MB Canada; 2 Department of Computer Science, University of Manitoba, Winnipeg, MB Canada; 3 Department of Chemistry, University of Manitoba, Winnipeg, MB Canada; 4 Department of Microbiology, University of Manitoba, Winnipeg, MB Canada; 5 Department of Medical Microbiology & Infectious Disease, University of Manitoba, Winnipeg, MB Canada; 6 Department of Biochemistry and Medical Genetics, University of Manitoba, Winnipeg, MB Canada; 7 Department of Biochemistry, Western University, Building Rm. 362, London, ON N6A 5C1 Canada

**Keywords:** Message-passing neural networks, Attention mechanism, Molecular representations, Atom-bond Transformer message-passing neural network, Molecular property prediction, Biological activity prediction

## Abstract

Graph convolutional neural networks (GCNs) have been repeatedly shown to have robust capacities for modeling graph data such as small molecules. Message-passing neural networks (MPNNs), a group of GCN variants that can learn and aggregate local information of molecules through iterative message-passing iterations, have exhibited advancements in molecular modeling and property prediction. Moreover, given the merits of Transformers in multiple artificial intelligence domains, it is desirable to combine the self-attention mechanism with MPNNs for better molecular representation. We propose an atom-bond transformer-based message-passing neural network (ABT-MPNN), to improve the molecular representation embedding process for molecular property predictions. By designing corresponding attention mechanisms in the message-passing and readout phases of the MPNN, our method provides a novel architecture that integrates molecular representations at the bond, atom and molecule levels in an end-to-end way. The experimental results across nine datasets show that the proposed ABT-MPNN outperforms or is comparable to the state-of-the-art baseline models in quantitative structure–property relationship tasks. We provide case examples of *Mycobacterium tuberculosis* growth inhibitors and demonstrate that our model's visualization modality of attention at the atomic level could be an insightful way to investigate molecular atoms or functional groups associated with desired biological properties. The new model provides an innovative way to investigate the effect of self-attention on chemical substructures and functional groups in molecular representation learning, which increases the interpretability of the traditional MPNN and can serve as a valuable way to investigate the mechanism of action of drugs.

**Supplementary Information:**

The online version contains supplementary material available at 10.1186/s13321-023-00698-9.

## Introduction

With the rapid development and expanding applications of artificial intelligence (AI) in academia and industry, molecular property prediction has played a fundamental role in the early stage of drug discovery. By training effective computational models and delivering accurate prediction of molecular properties, potential drug candidates were identified from virtual screening libraries of small molecules, thus addressing the intensive monetary investment and time-consuming nature of early stage drug discovery process [1–3]. In this context, expressive molecular representation modeling performed by a high-precision machine learning (ML) model is indispensable and has garnered significant attention from researchers.

Similar to convolutional neural networks (CNNs) that learn latent featurization of structural data by conducting convolutional operations, graph convolutional neural networks (GCNs) can generalize the convolutional operation to non-structural data and aggregate global information from local features. As small molecules can be naturally considered as graph data in the computational context, GCNs have been wildly applied to molecular property prediction tasks and have achieved remarkable success [4, 5]. The essence of graph convolution in the spatial domain is the process of designing node-level feature aggregation functions, so that information from the local neighborhoods of the nodes can be transmitted and aggregated throughout the graph [6, 7]. Among the variants of spatial-based GCNs, message-passing neural network (MPNN) [8] is a classic approach and outlines general frameworks for utilizing spatial graph convolutions.

The integration of the self-attention [9] mechanism into the message-passing neural network is of great interest as it can learn a better representation from molecular graphs. While the local information of molecules can be transmitted and aggregated within graphs without distance restrictions, every atom or bond has the same weight of impact on the predicted outcomes due to the averaging effect in graph convolution or message-passing schemes. However, in reality, each molecule forms a particular conformation in the 3D space to reach the minimum energy states. The molecular properties and mechanism of action (MOA) of specific molecules are critically governed by their conformations. Topologically adjacent or close atoms that are connected by bonds can potentially form functional groups or fragments that determine the properties of molecules, such as toxicity. By integrating attention mechanisms with MPNNs, the models can focus more on substructures critical to the desired chemical properties in the learning process, thus yielding more informative molecular representations.

Given the strengths and various successful practices of the Transformer models, several previous works have augmented self-attention to GCNs, whereas the majority of them employed the self-attention mechanism during the node (atom) embedding [10–12]. For example, Attentive FP proposed by Xiong et al. [13] extended a graph-based neural network with the self-attention on both atom and molecule embedding, where they treated the entire molecule as a super-virtual node connecting to all atoms. Although these models can learn expressive encodings of molecules by applying graph attention to atoms, none of them modeled the interactions of atomic bonds during the message-passing. In cheminformatics, besides the attention to the local environment of molecules, some studies have explored the self-attention mechanism in other aspects during representation learning. For instance, Chuang et al. [14] developed the attention mechanism on top of a GCN to aggregate results over molecular conformers. In their network, the attention coefficients are assigned to individual encodings of conformers, whereas the modeling of attention inside the molecular graphs is omitted.

In this work, we propose an Atom-Bond Transformer-based Message-passing Neural Network (ABT-MPNN), in which we adopted additive attention and scaled dot-product attention to the MPNN framework at both bond and atom levels, respectively. The additive attention [15] is an attention mechanism that is performed by calculating the attention alignment score of the hidden states of the encoder and decoder in the form of feed-forward layers. The scaled dot-product attention [9] is achieved by modeling the interaction between query and key through dot-product, followed by a scaling factor to scale down the results of dot-products. At the atom level attention, we further incorporate three types of inter-atomic feature matrices (atom and bond feature matrix, adjacency and distance matrix and coulomb matrix) into the model to provide structural and electrostatic information about molecules. Finally, we enable our model with the attention-based visualization modality on atoms using similarity maps [16], where topography-like molecular maps are colored based on the atomic contribution (weight) to the desired properties.

The novelty of this model can be summarized as follows: i) our work integrates the additive attention and the scaled dot-product attention into graph-based models and highlights the effect of self-attention on both atoms and bonds of molecules; ii) we introduce the Coulomb matrix to the network and design a feature-engineering scheme in which each attention head only comprises one type of scaled feature matrix in addition to the trained attention weights. This improvement is inspired by the Molecule Attention Transformer (MAT) proposed by Maziarka et al. [11], where adjacency and distance matrices were combined and added to every attention head.

## Materials and methods

### Preliminaries

We conduct a brief description of the preliminaries related to this work, including several graph-based molecular representations, message-passing neural networks, as well as the attention mechanism and Transformer.

#### Graph-based molecular representation

A graph $G$ is a data structure defined by a pair of sets $\left(V,E\right)$, where $V$ and $E$ represent the collections of vertices and edges, respectively. A directed graph has ordered pairs of vertices, where edges are directed from one vertex to another. In contrast, an undirected graph can be seen as a special case of directed graph in which elements of $E$ are unordered pairs of elements in $V$, meaning the edges between nodes have no direction associated with them. In modeling, the presence of a pair in $E$ (i.e., ${e}_{ij}=({v}_{i},{v}_{j})\in E$) signifies a specific connection between two vertices (i.e., ${v}_{i},{v}_{j}$) in $V$. While one may associate feature vectors to the elements in $V$ and/or those in $E$, these feature vectors are not strictly part of the graph data structure. Accordingly, a molecular graph comprises a set of atoms and a set of chemical bonds or interactions between each pair of adjacent atoms. Instead of characterizing the complete molecular information into a one-dimensional array such as molecular fingerprints, the graph structure permits association of a feature vector with each atom and with each bond. The graph-based representations can thus encode the properties or relationships of atoms and bonds locally with a collection of atom and bond feature vectors.

##### Atom and bond feature matrices

Various chemical properties can be calculated for atoms and bonds of molecules. The extracted atom and bond features are usually mapped into two-dimensional data arrays that can be easily handled by computers [17]. Specifically, an atom feature matrix can be generated by filling each row (representing each atom in the molecule) with atomic properties, such as atomic number, formal charge, and chirality. For a bond feature matrix, the values in each row correspond to attributes calculated for each bond in a molecule, which may include bond type, conjugation, ring membership, etc. In practice, categorical properties are commonly encoded in a one-hot manner to be more expressive.

##### Adjacency and distance matrices

Adjacency and distance matrices are two graph representations of molecules that contain the information of connectivity and distance for each pair of atoms, respectively. For an adjacency matrix, entries are set to 1 if chemical bonds exist between the corresponding atom pairs while nonbonded atom pairs are denoted with 0. In contrast to this binary definition of bonding, a distance matrix depicts the topological distances of atoms. For each molecule, a distance matrix is based on the molecular conformation and is calculated according to the 3D coordinates of atom pairs.

##### Coulomb matrix

The Coulomb matrix proposed by Rupp et al. [18] is a molecular featurization method that depicts the electrostatic interaction between atoms, which is specified by a set of nuclear charges {${Z}_{i}$} and the corresponding Cartesian coordinates {${R}_{i}$}. For each molecule, a Coulomb matrix is encoded by atomic energies and the inter-nuclear Coulomb repulsion operator as follows:

1 $${M}_{ij}=\left\{\begin{array}{c}0.5{Z}_{i}^{2.4}\, (i=j)\\ \frac{{Z}_{i}{Z}_{j}}{\left|{R}_{i}-{R}_{j}\right|} \,(i\ne j)\end{array}\right.$$

The elements on diagonal $(i=j)$ represent the interaction of atoms with themselves and are assigned with a polynomial fit of atomic energy. The rest of the entries $(i\ne j)$ are calculated by the Coulomb repulsion operator.

#### Message-passing neural networks

The MPNN proposed by Gilmer et al. [8] is another type of spatial-based approach that operates on undirected graphs with both node and edge features. The MPNN abstracts the commonalities of spatial convolutions and can be used as a general framework for spatial-based GCNs. The MPNN framework generally comprises two phases to obtain global graph features: a message-passing phase and a readout phase. Specifically, the message-passing phase consists of $T$ iterations to aggregate information for each node. A graph is first initialized by node features ${x}_{v}$ and edge features ${e}_{vw}$. In each message-passing step $t$ ($1\le t\le T$), the hidden representation (${h}_{v}^{t}$) and the message ${m}_{v}^{t}$ associated with each node *v* are updated at *t* + 1 according to

2 $${m}_{v}^{t+1}=\sum_{w\in N(v)}{M}_{t}({h}_{v}^{t},{h}_{w}^{t},{e}_{vw})$$

3 $${h}_{v}^{t+1}={U}_{t}({h}_{v}^{t},{m}_{v}^{t+1})$$

where ${M}_{t}$ is a message function and ${U}_{t}$ is a vertex update function. After $T$ iterations, the readout phase, with a readout function $R,$ is used to aggregate a global representation for the entire graph from all hidden representations of nodes as follows:

4 $${\widehat{y}=R(\{h}_{v}^{T}\left|v\in G\right\})$$

With different definitions of ${M}_{t}$, ${U}_{t}$, and $R$, multiple spatial-based GCNs can be generalized into the MPNN framework. The MPNN framework has been extensively used in computational chemistry and biology fields for modeling molecular structures due to the flexible and customizable message/update functions. For instance, a robust and powerful architecture called directed message-passing neural network (D-MPNN) [19] engineers message aggregation schemes associated with directed bonds rather than atoms. Using such a design, D-MPNN can avoid unnecessary loops and redundancies in the message-passing iterations, thus allowing effective aggregation of local information to the molecular level.

#### Attention mechanism and transformer

The Transformer [9], a new deep learning approach that uses the self-attention mechanism to differentially weigh the significance of each part of the input data and its variants, has emerged as one of the most potent architectures for modeling sequence data in natural language processing. Unlike the convolutional operation in the traditional convolutional neural network, the self-attention mechanism, which serves as the Transformer's core, can efficiently model the sequence data by capturing the interactions between each pair of input tokens. Transformer-like architectures have been applied and show great promise in multiple AI domains, such as vision Transformer, [20] developed for computer vision tasks, and AlphaFold2, [21] designed for protein folding problems.

The Transformer network [9] is built upon the self-attention mechanism, where a scaled dot-product scoring function is applied to model the context by capturing the correspondence between each pair of the position of the input. Specifically, a self-attention layer takes an input hidden matrix $H\in {\mathbb{R}}^{N\times d}$, where $N$ is the number of entries and $d$ is their hidden dimension. The input is projected to a query matrix (${Q=HW}_{Q}$), a key matrix (${K=HW}_{K}$) and a value matrix (${V=HW}_{V}$), where ${W}_{Q}$, ${W}_{K}$ and ${W}_{V}$ are the parameter matrices. The self-attention in the Transformer is computed as:

5 $$Attention(Q,K,V)=softmax(\frac{Q{K}^{T}}{\sqrt{d}})V$$

Instead of calculating a single attention function to the queries, keys, and values, the Transformer uses multi-head self-attention, where multiple attention functions are performed in parallel and then projected to form the overall output. Specifically, for each attention head (${head}_{i}$), the learned representation is formulated as:

6 $${head}_{i}=Attention\left(Q{W}_{{Q}_{i}},K{W}_{{K}_{i}},V{W}_{{V}_{i}}\right)=softmax(\frac{Q{W}_{{Q}_{i}}{(K{W}_{{K}_{i}})}^{T}}{\sqrt{d}})V{W}_{{V}_{i}}$$

where ${W}_{{Q}_{i}}$, ${W}_{{K}_{i}}$, ${W}_{{V}_{i}}$ are learnable weight matrices for ${head}_{i}$. Next, the outputs of attention heads are concatenated and projected by a parameter matrix ${W}_{O}$ to produce the final output:

7 $$MultiHead(Q,K,V)=Concat({head}_{1},\dots ,{head}_{h}){W}_{O}$$

### Atom-bond transformer-based message-passing neural network

#### Model architecture

**Fig. 1:**
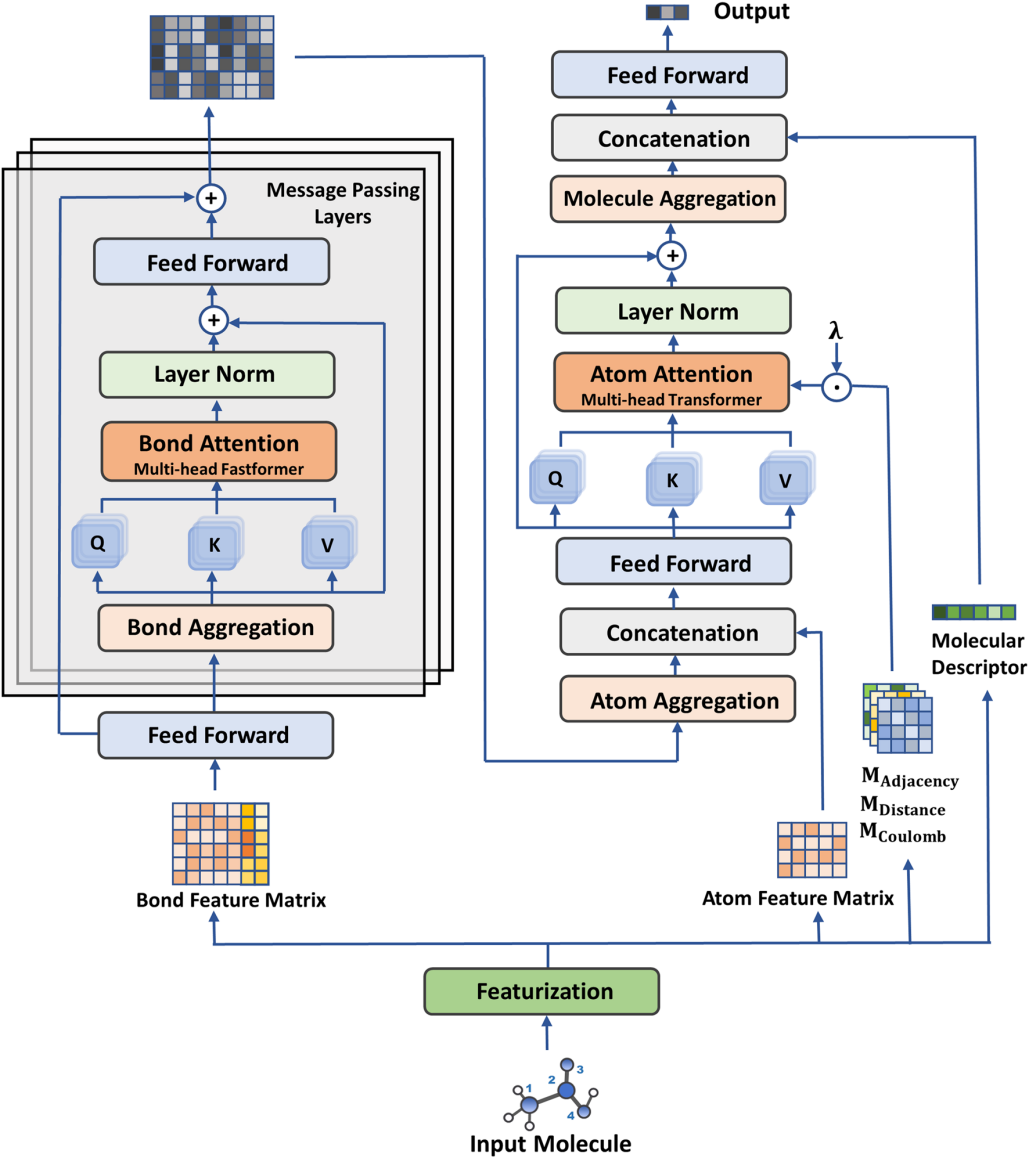
Illustration of our proposed ABT-MPNN. The given network takes the SMILES as input and generates atom features, bond features, three inter-atomic matrices and molecular descriptors as local and global encodings of the molecule. The bond feature matrix is first learned via bond attention blocks and bond update functions in the message-passing layers. After the message-passing phase, the atomic representations are obtained by summing the incoming bond hidden states, followed by the concatenation of the atom feature matrix and a multi-head atom attention block. In the atom attention block, three scaled inter-atomic matrices are individually added to each attention head’s weights as a bias term. Finally, the learned atomic hidden states are aggregated to a molecular vector, concatenated with the molecular descriptors, then entered into feed-forward layers for property prediction

The architecture of the proposed atom-bond Transformer-based message-passing neural network (ABT-MPNN) is shown in Fig. 1. As previously defined, the MPNN framework consists of a message-passing phase and a readout phase to aggregate local features to a global representation for each molecule. According to this paradigm, D-MPNN defines a novel message-passing phase through directed bonds. Here, we further extend D-MPNN by integrating the self-attention mechanism at the bond and atom levels with two Transformer-like architectures and design a feature engineering scheme at the atom attention step.

More concretely, molecules represented by the simplified molecular-input line-entry system (SMILES) are first entered into the featurization step, and node features (${x}_{v}$) and bond features (${e}_{vw}$) are generated, most of which are one-hot encoded (Additional file 1: Table S1). In addition, three inter-atomic (adjacency, distance, coulomb) matrices and a feature vector containing molecular descriptors (${h}_{f}$) are also generated (Additional file 1: Table S2). Since hidden states are transmitted in a directed manner in the message-passing (bond embedding) phase, each bond is initialized with two feature vectors, representing the bond messages in two opposite directions. Before the bond embedding stage, the hidden states for chemical bonds (${h}_{vw}^{0}$) are initialized, where ${W}_{i}$ is the first learnable weight matrix of the model (Table 1: Initialization).

**Table 1:** Algorithm of ABT-MPNN

Initialization	
1)	Given a molecular graph $G$, generate atom features ${x}_{v}$ and bond features ${e}_{vw}$ where $v\in Atom\left(G\right)$ and $w\in Neighbor\left(v\right)$; three inter-atomic matrices ${M}_{Adjacency},{M}_{Distance},{M}_{Coulomb}$; molecular descriptors ${h}_{f}$
2)	for each atom $v$ in molecule $G$:
3)	for each atom $w$ in molecule $Neighbor\left(v\right)$:
4)	$\,\,\,\,\,\,\,\,{h}_{vw}^{0}\leftarrow ReLU({W}_{i}Concat\left({x}_{v},{e}_{vw}\right))$

Bond Embedding Phase	
1)	Message-passing iteration:$t=1, 2,\dots ,T$
2)	while $1\le t\le T$:
3)	for each atom $v$ in molecule $G$:
4)	for each atom $w$ in molecule $Neighbor\left(v\right)$:
5)	$\,\,\,\,\,\,\,\,\,\,\,\,{m}_{vw}^{t}\leftarrow \sum_{k\in Neighbor\left(v\right)}{h}_{kv}^{t-1}-{h}_{wv}^{t-1}$
6)	$\,\,\,\,\,\,\,\,\,\,\,\,{b}_{vw}^{t}\leftarrow BondAttention\left({m}_{vw}^{t}\right)+{m}_{vw}^{t}$
7)	$\,\,\,\,\,\,\,\,\,\,\,\,{h}_{vw}^{t}\leftarrow ReLU({h}_{vw}^{0}+{W}_{h} {b}_{vw}^{t})$

Atom Embedding Phase	
1)	for each atom $v$ in molecule $G$:
2)	$\,\,\,\,{m}_{v}\leftarrow ReLU({W}_{o}Concat({x}_{v},\sum_{w\in Neighbor\left(v\right)}{h}_{vw}^{T}))$
3)	$\,\,\,\,{h}_{v}\leftarrow AtomAttention\left({m}_{v},{M}_{Adjacency},{M}_{Distance},{M}_{Coulomb }\right)+{m}_{v}$

Molecule Embedding Phase	
1)	$h\leftarrow \sum_{v\in G}{h}_{v}$
2)	$\widehat{y}\leftarrow FFN(Concat(h,{h}_{f}))$

At each message-passing iteration $t$, each bond message (${m}_{vw}^{t}$) is first updated by summing all the incoming neighboring hidden states (${h}_{kv}^{t-1},k\in Neighbor\left(v\right)$) from the previous iteration, except the one that represents the opposite direction of its own (${h}_{wv}^{t-1}$). Next, we augment a multi-head self-attention to the bond messages and add the input bond messages to the bond attention output through a skip connection. Specifically, to produce the attention for each bond, the bond attention block takes in all the bond messages from the previous message-passing iteration as input. The obtained bond attention message (${b}_{vw}^{t}$) is projected by a hidden weight matrix (${W}_{h}$), concatenated with the original bond hidden state (${h}_{vw}^{0}$), then fed into an activation function to generate the hidden state (${h}_{vw}^{t}$) that is used for the following message-passing iteration. Compared with the generic message-passing scheme described in the previous section, the employment of bond attention has an additional step of updating the hidden representation (${h}_{vw}^{t}$) (Table 1: Bond Embedding Phase).

After iterating through all the message-passing layers, the message of each atom (${m}_{v}$) is obtained by aggregating all the adjacent bond hidden states that originated from it (${h}_{vw}^{T},w\in Neighbour\left(v\right)$) and concatenating them with atom features, which are then transformed by a weight matrix (${W}_{o}$) and a ReLu activation. Here, we further implement an atom-level Transformer block assisted with three atom-wised matrices and a skip connection from the input to generate the hidden states for atoms (Table 1: Atom Embedding Phase). At the molecule embedding phase, all the learned atomic hidden states of a molecule are summed together as a single representation ($h$). The final output of the model is returned by a two-layer feed-forward neural (FFN) network that is fed with the concatenation of the learned representation and the calculated molecular descriptors (Table 1: Molecule Embedding Phase).

#### Bond attention

Prior to the scaled dot-product attention used in the Transformer network, the additive attention proposed by Bahdanau et al. [15] is known as the earliest attempt to use the attention mechanism in deep learning. Based on the additive attention, Wu et al. [22] proposed an efficient Transformer architecture, namely Fastformer, to mitigate the quadratic computational complexity in the Transformer network. In general, instead of modeling the interactions between each pair of units by dot-product of matrices, Fastformer uses additive attention to model global contexts and transform each token representation by its interaction with the global contexts. Since the MPNN framework contains $T$ message-passing iterations, adding the Transformer architecture in each message-passing layer is computationally expensive, especially for architectures containing numerous layers to train large molecules. To this end, we adopt Fastformer as the building block for bond attention in our model.

The pseudo-code of the bond attention is shown in Additional file 1: Table S3. Specifically, the bond attention block contains 6 attention heads and takes the bond messages as input. Given a molecule with $N$ bonds, the query, key, and value matrices are set equal to the input bond message matrix ${H}_{b}\in {\mathbb{R}}^{2N\times d}$, where $d$ is the hidden dimension. Firstly, a global bond query (${q}_{b}$) is obtained via the additive attention, in which an additive attention weight (${\alpha }_{{b}_{i}}$) of each bond vector is calculated, multiplied by its corresponding bond query vector (${q}_{{b}_{i}}$) and summarized together. Next, the interaction between the global bond query and the bond key vectors (${k}_{{b}_{i}}$) is carried out by element-wise products. Similarly, a global bond key (${k}_{b}$) is obtained by conducting additive attention and is employed to transform the bond value vectors by element-wise products. Lastly, the resulting key-value interaction vectors are projected, added with the bond queries (${q}_{{b}_{i}}$) through a skip connection, then normalized by a layer normalization [23] to generate the final bond attention output ${O}_{b}\in {\mathbb{R}}^{2N\times d}$.

#### Atom attention

At the atom embedding phase, we further construct a multi-head self-attention layer on the aggregated atom vectors, allowing the model to focus more on atoms or local environments that are most relevant to the target properties. Instead of using additive attention, we select the original Transformer network that uses scaled dot-product attention as our building block for atom attention. The motivation for this choice is mainly due to the encapsulation of additional features. Concretely, due to the architectural constraints, most graph-based networks only operate on molecular graphs where atoms or bonds are embedded with feature vectors containing corresponding chemical properties. With the inclusion of scaled dot-product attention on atoms, our model can incorporate additional graph-level features that contain information on the spatial and electrostatic relationships between pairs of atoms, thus providing a more comprehensive perspective from molecular topology during modeling.

As defined in Additional file 1: Table S4, the atom attention layer with 6 attention heads takes the aggregated atom messages (${H}_{a}\in {\mathbb{R}}^{M\times d}$) as input, where $M$ is the number of atoms and $d$ is the hidden dimension. For each attention head, one type of additional inter-atomic feature matrix is added to the query-key interaction matrix as a bias term. Specifically, the ${head}_{1}$ and ${head}_{2}$ take the adjacency matrices of molecules as inputs, which incorporates the connectivity information of molecules into the model. The ${head}_{3}$ and ${head}_{4}$ include the topological distances of atom pairs from the RDKit generated conformers to the attention weights. The ${head}_{5}$ and ${head}_{6}$ encapsulate the Coulomb matrix, which depicts the electrostatic interaction between atoms in the model. Before importing them to the model, the feature matrices are normalized by Z-score normalization and scaled by $\lambda$, a hyperparameter used in this architecture.

### Experimental settings

#### Benchmark datasets and evaluation metrics

As an extension of our previous framework for modeling large-scale chemical-genetic datasets, we conducted the performance evaluation of the proposed ABT-MPNN on the chemical-genetic interaction profiles of drugs from Johnson et al. [24], which include 47,217 small molecules against hundreds of *Mycobacterium tuberculosis* mutant strains (named by the downregulated gene). The growth inhibition property of a molecule on each *M. tuberculosis* mutant strain was gauged by the statistical test (Z-score) obtained from the experimental results [24]. The smaller the Z-score, the more pronounced the growth inhibitory effect of the small molecule on the *M. tuberculosis* mutant strain. We later clustered the chemical-genetic interaction profiles in gene clusters by first identifying *M. tuberculosis* H37Rv homologs in *Escherichia coli* K12 according to their gene products. Then, the semantic gene similarity of biological process for the homologs were calculated and hierarchical clustering was performed [25]. After the gene-level clustering, 13 distinct *M. tuberculosis* gene groups were formed, and the target value for each gene cluster was obtained by finding the median Z-score of the genes in that cluster. Besides training regression models with continuous Z-scores, we built binary classification tasks for each of the 13 gene clusters with a class criterion equal to -4, where Z-score < −4 was considered growth inhibitory or active (1), or otherwise inactive (0). For this dataset (Table 2), we employed a random split to divide the data into subsets (training set, validation set, and test set) by the ratio of 80:10:10. Root mean squared error (RMSE) was used as the metric for regression and the area under the precision-recall curve (AUPRC) was used for classification since the binarized dataset is highly imbalanced (the average percentage of positive labels across clusters is 4%).

**Table 2:** The summary of the selected molecular datasets

Task type	Dataset	No. tasks	No. compounds	Data split	Metric
Classification	Johnson et. al	13	47,217	Random	AUPRC
	Tox21	12	7,831	Random	AUROC
	ClinTox	2	1,478	Random	AUROC
	ToxCast	617	8,576	Random	AUROC
	HIV	1	41,127	Scaffold	AUROC
Regression	Johnson et. al	13	47,217	Random	RMSE
	QM8	12	21,786	Random	MAE
	ESOL	1	1128	Random	RMSE
	FreeSolv	1	642	Random	RMSE
	Lipophilicity	1	4,200	Random	RMSE

In addition, we conducted prediction of molecular properties using 4 classification and 4 regression molecular benchmarks from MoleculeNet [26] (Table 2). We followed the recommendations of MoleculeNet [26] for selecting data split strategies and evaluation metrics, which were based on the content of each dataset and previous works. The Scaffold split was employed on the HIV dataset, while the rest used random split as default. The area under the receiver operating characteristic curve (AUROC) was applied to the 4 classification datasets. RMSE was calculated for regression tasks on ESOL, FreeSolv, and Lipophilicity, while mean absolute error (MAE) was applied to QM8.

#### Baseline models

We performed comparative evaluations of ABT-MPNN against 6 baseline methods covering shallow and deep ML architectures. These include (1) Random forest (RF) [27] with binary Morgan fingerprints as inputs; (2) feed-forward network (FFN) trained with normalized chemical descriptors. As our model was derived from the MPNN framework, we also reported the performance of (3) the message-passing neural network (MPNN) [8] and (4) the directed message-passing neural network (D-MPNN) [19] in the results. Additinoally, we compared our model with two other state-of-the-art graph neural networks: (5) DeeperGCN [28] and (6) geometry-enhanced molecular representation learning method (GEM) [29], to demonstrate the power of our proposed approach.

#### Implementation details

The RF was implemented with 500 trees based on binary Morgan fingerprints ($r=2$; $bits=2048$). The FFN contained a dense layer with 1400 neurons before the output layer and was fed with 200 normalized chemical descriptors. To improve models’ performance, the hyperparameters of models were optimized by Bayesian optimization [30] with the same optimization budget (30 epochs in 20 iterations) on the same data split. For our proposed model, we optimized the four hyper-parameters listed in Table 3.

**Table 3:** Bayesian Optimization for Hyperparameters in ABT-MPNN

Hyperparameters	Values
Message-passing iteration (T)	2, 3, 4, 5, 6
Inter-atomic feature scaler (λ)	[0, 0.5] (Interval: 0.05)
Hidden dimension (d)	[300, 2400] (Interval: 100)
Dropout probability (p)	[0, 0.4] (Interval: 0.05)

The models were optimized with the Adam optimizer, and the optimum parameters were determined as the ones with the highest performance score on the validation set during training. We employed a fivefold cross-validation (CV) on the partitioned data splits and reported the mean and standard deviation of the metrics. The ABT-MPNN used PyTorch [31] as the deep learning framework and was developed based on the Chemprop package by Yang et al. [32].

## Results and discussion

### Performance comparison with baselines

We compared our proposed ABT-MPNN with 6 baseline models on 10 classification and regression tasks, covering chemical-genetic interaction profiles (Johnson et al. [24, 25]) and a wide range of molecular properties in the field of quantum mechanics (QM8 [33]), physical chemistry (ESOL [34], lipophilicity [35], hydration free energies (Freesolv [36]), biophysics (HIV [26]), and physiology (Tox21 [37], Clintox [38], ToxCast [39]). The overall performance of a model on each dataset is represented as the mean $\pm$ standard deviation of the evaluation metrics across a fivefold CV, as shown in Table 4. From the results, ABT-MPNN achieved the best performance on all classification datasets, except on Tox21 where GEM provided the leading performance. Specifically, the Johnson et al. (classification) dataset achieved 4.98% performance increase compared to the second-best model D-MPNN. According to Clintox, ToxCast, and HIV, ABT-MPNN obtained 1.01%, 0.40% and 0.75% relative improvements compared to the second-ranked model, respectively. For classification, the result of RF on the ToxCast dataset is omitted due to high computational costs with 617 individual tasks.

**Table 4:** The performance comparison for classification and regression tasks

**Classification** (the higher the better)^a^					
	Johnson et al	Tox21	Clintox	ToxCast	HIV
RF	0.252 $\pm$ 0.014	0.818 $\pm$ 0.005	0.721 $\pm$ 0.088	_^b^	0.798 $\pm$ 0.040
FFN	0.258 $\pm$ 0.015	0.837 $\pm$ 0.010	0.837 $\pm$ 0.062	0.738 $\pm$ 0.009	0.803 $\pm$ 0.045
MPNN	0.258 $\pm$ 0.013	0.859 $\pm$ 0.011	0.873 $\pm$ 0.051	0.752 $\pm$ 0.010	0.788 $\pm$ 0.050
D-MPNN	0.281 $\pm$ 0.028	0.855 $\pm$ 0.015	0.895 $\pm$ 0.037	0.749 $\pm$ 0.013	0.788 $\pm$ 0.039
Deeper GCN	0.272 $\pm$ 0.022	0.853 $\pm$ 0.013	0.870 $\pm$ 0.042	0.751 $\pm$ 0.010	0.789 $\pm$ 0.031
GEM	0.280 $\pm$ 0.018	**0.864 **$\pm$** 0.010**	0.825 $\pm$ 0.091	0.757 $\pm$ 0.013	0.769 $\pm$ 0.038
ABT-MPNN	**0.295** $\pm$ **0.021**	0.857 $\pm$ 0.010	**0.904** $\pm$ **0.034**	**0.760** $\pm$ **0.013**	**0.809** $\pm$ **0.036**

Regression (the lower the better)^a^					
	Johnson et al	ESOL	Lipophilicity	Freesolv	QM8
RF	1.315 $\pm$ 0.021	1.230 $\pm$ 0.066	0.846 $\pm$ 0.039	2.467 $\pm$ 0.570	0.014 $\pm$ 0.000
FFN	1.321 $\pm$ 0.016	0.614 $\pm$ 0.109	0.674 $\pm$ 0.043	1.275 $\pm$ 0.352	0.016 $\pm$ 0.000
MPNN	1.309 $\pm$ 0.017	0.575 $\pm$ 0.086	0.585 $\pm$ 0.044	1.042 $\pm$ 0.220	0.010 $\pm$ 0.000
D-MPNN	1.307 $\pm$ 0.024	0.594 $\pm$ 0.066	0.558 $\pm$ 0.044	0.915 $\pm$ 0.142	0.010 $\pm$ 0.000
Deeper GCN	1.325 $\pm$ 0.015	0.601 $\pm$ 0.056	0.580 $\pm$ 0.035	0.970 $\pm$ 0.368	0.012 $\pm$ 0.000
GEM	1.315 $\pm$ 0.021	0.632 $\pm$ 0.062	0.599 $\pm$ 0.035	0.962 $\pm$ 0.257	0.010 $\pm$ 0.000
ABT-MPNN	**1.305 **$\pm$** 0.017**	**0.566** $\pm$ **0.075**	**0.554** $\pm$ **0.041**	**0.902 **$\pm$** 0.157**	**0.009** $\pm$ **0.000**

^a^The evaluation metrics are represented as averaged values ± standard deviation from fivefold CV. The best performance values are highlighted in bold

^b^The results of RF on ToxCast are not presented because of the substantial computational cost

Regarding regression tasks, we observed that the ABT-MPNN model achieved substantial improvements over classification, as it consistently outperformed all baseline models according to the results of the fivefold CV. The outstanding performance of ABT-MPNN on regression datasets could be associated with the modeling of inter-atomic attention with topological and electrostatic features, as regression tasks focus on linking quantum chemical properties to molecular structures, in which such information is of high relevance. In regression tasks of the Johnson et al. dataset, the ABT-MPNN model improved upon D-MPNN by a modest margin of 0.15%, and it boosted the results of QM8 with a 10% relative MAE optimization compared to MPNN, D-MPNN and GEM. Moreover, our model yielded superior results in RMSE compared to the second-best baselines on ESOL (1.57%), Freesolv (1.42%), and Lipophilicity (0.72%), respectively.

Overall, ABT-MPNN achieved state-of-the-art results on 9 out of 10 classification and regression tasks according to the fivefold CV, showing the robustness of the molecular representation learned by our model. The superior performances across multiple datasets compared to D-MPNN further support the effectiveness of complementing the directed message-passing scheme with the bond and atomic level attention.

### Ablation study

To validate the impact and contribution of each component to the performance of the proposed ABT-MPNN, we conducted a series of ablation studies on both classification (ClinTox) and regression (ESOL) datasets from our benchmarks. For each run, we kept the same hyper-parameter settings, and the performance was evaluated on the same fivefold CV, as is shown in Table 5. To better evaluate the results, Additional file 1: Fig. S1 shows the score for each ablation experiment on individual fold. Following the architecture design of the ABT-MPNN, we focused on investigating two key components of our model: bond attention and atom attention.

**Table 5:** Ablation study results on classification (ClinTox) and regression (ESOL) tasks

No	Bond attention		Atom attention		Classification (ClinTox)	Regression (ESOL)
	Transformer	Fastformer	No inter-atomic matrices	With inter-atomic matrices		
1					0.890 $\pm$ 0.040	0.582 $\pm$ 0.070
2	√				0.887 $\pm$ 0.044	0.570 $\pm$ 0.070
3		√			0.894 $\pm$ 0.042	0.573 $\pm$ 0.066
4			√		0.896 $\pm$ 0.035	0.569 $\pm$ 0.065
5				√	**0.905** $\pm$ **0.041**	0.569 $\pm$ 0.065
6		√	√		**0.905** $\pm$ **0.028**	0.567 $\pm$ 0.066
7		√		√	0.904 $\pm$ 0.034	**0.566 **$\pm$** 0.075**

The evaluation metrics are represented as averaged values ± standard deviation from fivefold CV. The best performance values are highlighted in bold

#### Effect of bond attention in the message-passing phase

One of the most important distinctions between ABT-MPNN and previous works is the integration of bond-level attention during the message-passing phase. In ABT-MPNN, we chose Fastformer [22] as the building block of the bond attention, given that it uses additive attention to model the global bond context, enabling effective representational modeling while mitigating high computational complexity. To verify the expressive power of the Fastformer approach, we also implemented Transformer in the message-passing phase as the bond attention block and conducted experiments #1, #2 and #3 for comparison (Table 5). From the experiments, the bond attention scheme improved the performance of baseline #1, which does not apply bond attention except for the inclusion of Transformer, which slightly reduced the performance of the classification. Regarding individual folds of the ClinTox dataset (Additional file 1: Fig. S1), the employment of bond attention improved or achieved on-par performance compared to baseline #1, except for fold 2 and fold 3. In comparison between two types of attention mechanism, Fastformer exceeded Transformer on three folds but Transformer got the highest AUROC score among all the experiments on fold 1. Regarding regression, both Transformer and Fastformer considerably enhanced the performance in general. Specifically, the bond-level attention, regardless of the architecture of the attention block, consistently improved the baseline on four data folds. Between the two attention architectures, Transformer achieved a modestly better performance than Fastformer. Possibly, the scaled dot product attention models a better bond-level representation in specific regression tasks than the additive attention developed in Fastformer. However, considering the superior performance of Fastformer on classification tasks and linear complexity of computing attention, we chose Fastformer as the building block of bond attention in ABT-MPNN.

#### Contribution of atom attention and inter-atomic features

As introduced in the methods section, we constructed atom-level self-attention with Transformer and incorporated additional adjacency, distance, and coulomb matrices into each attention head as auxiliaries. We analyzed the choices and optimizations at the atom embedding phase (Table 5) in experiments #4, #5 and baseline #1. Notably, the model showed marked improvements after employing atom attention, regardless of whether the additional inter-atomic matrices were included. This finding implies that the atomic-level self-attention facilitates representation learning by assigning more attention (weights) to the atoms or molecular functional groups that contribute to the property of interest. We will further examine this in our discussion section. Interestingly, through comparison of #4 and #5, the inter-atomic matrices increased the AUROC score of Clintox while on ESOL the model with this setting was insensitive to the topological and electrostatic information as they achieved identical RMSE scores. From the observation in Additional file 1: Fig. S1, the differences in the exclusion/inclusion of inter-atomic matrices on the regression task are marginal, in which two folds (3, 4) obtained smaller RMSE scores with the three inter-atomic matrices while three folds (1, 2, 5) did not.

#### Combination of bond attention and atom attention

Finally, we evaluated the effect of using bond attention and atom attention to justify our architecture design. Specifically, model #7 (Table 5) is the complete ABT-MPNN where it incorporates Fastformer-based bond attention and Transformer-based atom attention with inter-atomic features. In contrast to #7, the inter-atomic features in experiment #6 were excluded. Comparing #6 with experiments #3 and #4, in which bond and atomic level attentions were employed separately, we observed that combining attentions at the atomic and bond levels boosted the performance in both classification and regression. With respect to each fold, the combination of bond attention and atom attention (#6) resulted in a substantial increase in AUROC scores for folds 1, 2, and 4. For regression, although experiment #6 exhibited better mean RMSE than #3 and #4, the advantage of adopting atom and bond level attention together was not as pronounced as for classification due to the large deviation of results on the CV folds. From experiments #6 and #7, although the inclusion of the inter-atomic matrices marginally reduced the performance of classification by 0.11%, it further optimized the results of regression and achieved the best results among all the ablation experiments.

### Interpretability and visualization

Besides assessing the model’s performance, it is often beneficial to look into the “black box” of the trained model and have a deeper understanding of which substructures of molecules contribute more to the compound activities/properties. With the interpretability of attention weights of atoms, it is possible to investigate the latent linkage between the molecular substructure and the predicted outcomes. Here, we visualize the atomic attention weights using the similarity map [16] (or predicted probability map in this case) implemented in RDKit.

**Fig. 2:**
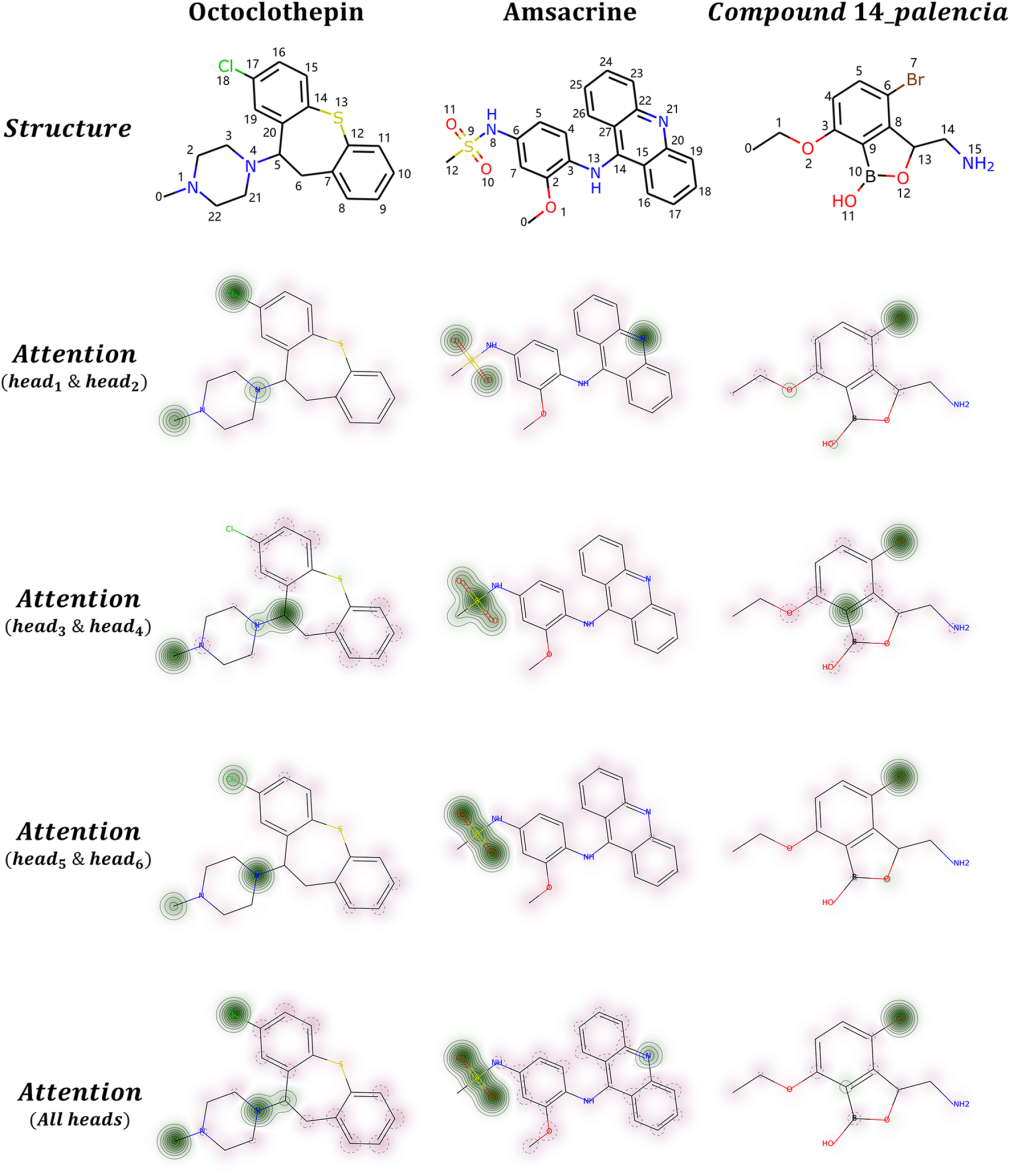
Visualization of the multi-head atom attention weights of the three *M. tuberculosis* growth inhibitors. In the predicted probability maps, atoms with positive contributions are colored in green, while red indicates that the corresponding attention weight is negative. The larger the absolute value, the darker the color shown on the map

Figure 2 visualizes the attention weights of different heads of three examples of anti-*M. tuberculosis* investigational drugs (Octoclothepin [40]; Amsacrine [41]; Compound 14_palencia [42]) curated from the study [25]. The compounds were chosen on the basis of 1) whole-cell inhibitory activity against wild-type *M. tuberculosis* or *M. smegmatis* and 2) biochemical validation of the molecular targets. Specifically, Nisa et al. [40] reported that Octoclothepin, an antipsychotic of the tricyclic group, exhibited inhibition of the in vitro ATPase activity of ParA from *M. tuberculosis*. Amsacrine is an antineoplastic agent that has been shown to inhibit mycobacterial *TopA*, the essential topoisomerase I involved in mycobacterial cell viability [41]. Compound 14, a potent *M. tuberculosis* protein synthesis inhibitor [42], can form adducts with AMP and together bind the ATPase pocket to inhibit the *LeuS* gene. Since we added an adjacency matrix to the ${head}_{1}$ and ${head}_{2}$, a distance matrix to the ${head}_{3}$ and ${head}_{4}$, and a Coulomb matrix to the ${head}_{5}$ and ${head}_{6}$, we followed this paradigm and visualized their averaged attention weights on rows 2–4 of the Fig. 2. The overall attention weights of the 6 attention heads are displayed in the last row.

First of all, we observe that the atom attention layer only focuses on a few atoms or substructures of the molecule and different attention branches have different “views” of the input. For instance, the weights in the ${head}_{1} \& {head}_{2}$ of Octoclothepin focus more on the chlorine ($Cl:\#18$) atom, while one of the nitrogen atoms ($N:\#4$) is assigned more attention weights in the ${head}_{5} \& {head}_{6}$. This observation demonstrates that the multi-head attention can give the architecture multiple subspaces to model the molecular representation regardless of training with the same input molecule. In addition, it is notable that most carbon ($C$) atoms of the three inhibitors gain attention values near zero, while green areas usually appear on the halogens or chalcogens that the inhibitors uniquely have. Furthermore, we observe that the attention mechanism of the ABT-MPNN facilitates the representation learning to the molecular functional groups. For instance, the results of Amsacrine demonstrate that all attention heads have emphasized the sulfonamide to varying degrees. Therefore, it is reasonable to speculate that the inhibitory capacity of Amsacrine against *M. tuberculosis* might be associated with its sulfonamide functional group, in agreement with the suggested interaction between the sulfonamide moiety and the mycobacterial topoisomerase I TopA [41].

## Conclusion

In this study, we proposed a novel message-passing framework called ABT-MPNN that incorporates both additive attention and scaled dot-product attention at the bond and atomic levels, respectively. To incorporate the topological and electrostatic information of molecules into the model, we further designed a feature engineering scheme that embedded adjacency, distance and Coulomb matrices derived from molecular conformations with each atom attention head.

Overall, our proposed model consistently outperformed or is comparable with the state-of-the-art baseline models on a wide range of molecular datasets. By introducing the attention schemes at the atomic level, we realized the visualization modality of the model via the predicted probability map. Through the demonstration of the three *M. tuberculosis* inhibitors, we highlighted the effect of self-attention on chemical substructures and functional groups during molecular representation learning, which not only increases the interpretability of the MPNN but also serves as a valuable way to investigate the mechanism of action.

## Supplementary Information

**Additional file 1:** **Table S1.** Atom and bond features. **Table S2.** 200 Molecular descriptors generated by RDKit. **Table S3.** Algorithm of Bond Attention. **Table S4. **Algorithm of Atom Attention. **Fig. S1. **Comparison of ablation experiments using 5-fold cross-validation (A) Performance evaluation of each fold for the classification task (ClinTox) measured with AUROC. Experiments settings: #1: baseline; #2: use bond attention (Transformer); #3: use bond attention (Fastformer); #4 use atom attention; #5 use atom attention with inter-atomic matrices #6 use bond attention (Fastformer) and atom attention; #7 use bond attention (Fastformer) and atom attention with inter-atomic matrices (B) Performance evaluation of each fold for the regression task (ESOL) measured by RMSE. The settings of each experiment in the regression task are identical to those in the classification one.

## Data Availability

The raw data from the Johnson et al. study is publicly accessible on the website: https://www.chemicalgenomicsoftb.com/. The scripts, datasets, and results supporting the conclusions of this article are available in the supplementary materials and our GitHub repository: https://github.com/LCY02/ABT-MPNN.

## Abbreviations

ABT-MPNN: Atom-bond Transformer-based message-passing neural network
AUPRC: Area under the curve of the precision-recall cure
AUROC: Area under the curve of the receiver operating characteristic curve
CV: Cross-validation
D-MPNN: Directed message-passing neural network
FFN: Feed-forward network
GCN: Graph convolutional neural network
ML: Machine learning
GEM: Geometry-enhanced molecular representation learning method
MAE: Mean absolute error
MOA: Mechanism of action
MPNN: Message-passing neural networks
M. tuberculosis: Mycobacterium tuberculosis
QSAR: Quantitative structure–activity relationship
QSPR: Quantitative structure–property relationship
RF: Random forest
RMSE: Root mean squared error
SMILES: Simplified molecular input line entry system
