# Supplementary material for: ABT-MPNN: an atom-bond transformer-based message-passing neural network for molecular property prediction
Source: J Cheminform. 2023 Feb 26;15:29. doi: 10.1186/s13321-023-00698-9 (PMC9968697; doi:10.1186/s13321-023-00698-9)

# Supporting Information

## A. Details about molecular features

**Table S1: Atom and bond features**

| Feature Type | Attribute | Size | Description |
|---|---|---|---|
| **Atom Features** | Atom type | 100 | Chemical elements with atomic number ≤ 100 |
| | Degree | 6 | No. of bonds in which the atom is involved |
| | Formal charge | 5 | Electronic charge assigned to an atom |
| | Chirality | 4 | Unspecified, tetrahedral CW/CCW, or other types of chirality |
| | Number of H | 5 | No. of bonded hydrogen atoms |
| | Hybridization | 5 | sp, $sp^2$, $sp^3$, $sp^3d$, or $sp^3d^2$ |
| | Aromaticity | 1 | Whether the atom is a component of an aromatic system |
| | Atomic mass | 1 | Mass of the atom (divided by 100) |
| **Bond Features** | Bond type | 4 | Single, double, triple, or aromatic |
| | Conjugated | 1 | Whether the bond is conjugated |
| | Ring | 1 | Whether the bond is in a ring |
| | Stereo | 6 | Stereochemistry of bonds (none, any, E/Z or cis/trans) |

**Table S2: 200 Molecular descriptors generated by RDKit**

| Module | Descriptor Name | | | |
|---|---|---|---|---|
| **Graph Descriptors** | BalabanJ | BertzCT | Chi0 | Chi0n |
| | Chi0v | Chi1 | Chi1n | Chi1v |
| | Chi2n | Chi2v | Chi3n | Chi3v |
| | Chi4n | Chi4v | HallKierAlpha | Ipc |
| | Kappa1 | Kappa2 | Kappa3 | |
| **Descriptors** | ExactMolWt | FpDensityMorgan1 | FpDensityMorgan2 | FpDensityMorgan3 |
| | HeavyAtomMolWt | MaxAbsPartialCharge | MaxPartialCharge | MinAbsPartialCharge |
| | MinPartialCharge | MolWt | NumRadicalElectrons | NumValenceElectrons |
| **Lipinski** | FractionCSP3 | HeavyAtomCount | NHOHCount | NOCount |
| | NumAliphaticCarbocycles | NumAliphaticHeterocycles | NumAliphaticRings | NumAromaticCarbocycles |
| | NumAromaticHeterocycles | NumAromaticRings | NumHAcceptors | NumHDonors |
| | NumHeteroatoms | NumRotatableBonds | NumSaturatedCarbocycles | NumSaturatedHeterocycles |
| | NumSaturatedRings | RingCount | | |
| **EState_VSA** | EState_VSA1 | EState_VSA10 | EState_VSA11 | EState_VSA2 |
| | EState_VSA3 | EState_VSA4 | EState_VSA5 | EState_VSA6 |
| | EState_VSA7 | EState_VSA8 | EState_VSA9 | VSA_EState1 |
| | VSA_EState10 | VSA_EState2 | VSA_EState3 | VSA_EState4 |

| | | | | |
|---|---|---|---|---|
| | VSA_EState5 | VSA_EState6 | VSA_EState7 | VSA_EState8 |
| | VSA_EState9 | | | |
| **EState** | MaxAbsEStateIndex | MaxEStateIndex | MinAbsEStateIndex | MinEStateIndex |
| **Crippen** | MolLogP | MolMR | | |
| **MolSurf** | LabuteASA | PEOE_VSA1 | PEOE_VSA10 | PEOE_VSA11 |
| | PEOE_VSA12 | PEOE_VSA13 | PEOE_VSA14 | PEOE_VSA2 |
| | PEOE_VSA3 | PEOE_VSA4 | PEOE_VSA5 | PEOE_VSA6 |
| | PEOE_VSA7 | PEOE_VSA8 | PEOE_VSA9 | SMR_VSA1 |
| | SMR_VSA10 | SMR_VSA2 | SMR_VSA3 | SMR_VSA4 |
| | SMR_VSA5 | SMR_VSA6 | SMR_VSA7 | SMR_VSA8 |
| | SMR_VSA9 | SlogP_VSA1 | SlogP_VSA10 | SlogP_VSA11 |
| | SlogP_VSA12 | SlogP_VSA2 | SlogP_VSA3 | SlogP_VSA4 |
| | SlogP_VSA5 | SlogP_VSA6 | SlogP_VSA7 | SlogP_VSA8 |
| | SlogP_VSA9 | TPSA | | |
| **Fragments** | fr_Al_COO | fr_Al_OH | fr_Al_OH_noTert | fr_ArN |
| | fr_Ar_COO | fr_Ar_N | fr_Ar_NH | fr_Ar_OH |
| | fr_COO | fr_COO2 | fr_C_O | fr_C_O_noCOO |
| | fr_C_S | fr_HOCCN | fr_Imine | fr_NH0 |
| | fr_NH1 | fr_NH2 | fr_N_O | fr_Ndealkylation1 |
| | fr_Ndealkylation2 | fr_Nhpyrrole | fr_SH | fr_aldehyde |
| | fr_alkyl_carbamate | fr_alkyl_halide | fr_allylic_oxid | fr_amide |
| | fr_amidine | fr_aniline | fr_aryl_methyl | fr_azide |
| | fr_azo | fr_barbitur | fr_benzene | fr_benzodiazepine |
| | fr_bicyclic | fr_diazo | fr_dihydropyridine | fr_epoxide |
| | fr_ester | fr_ether | fr_furan | fr_guanido |
| | fr_halogen | fr_hdrzine | fr_hdrzone | fr_imidazole |
| | fr_imide | fr_isocyan | fr_isothiocyan | fr_ketone |
| | fr_ketone_Topliss | fr_lactam | fr_lactone | fr_methoxy |
| | fr_morpholine | fr_nitrile | fr_nitro | fr_nitro_arom |
| | fr_nitro_arom_nonortho | fr_nitroso | fr_oxazole | fr_oxime |
| | fr_para_hydroxylation | fr_phenol | fr_phenol_noOrthoHbond | fr_phos_acid |
| | fr_phos_ester | fr_piperdine | fr_piperzine | fr_priamide |
| | fr_prisulfonamd | fr_pyridine | fr_quatN | fr_sulfide |
| | fr_sulfonamd | fr_sulfone | fr_term_acetylene | fr_tetrazole |
| | fr_thiazole | fr_thiocyan | fr_thiophene | fr_unbrch_alkane |
| | fr_urea | | | |
| **QED** | qed | | | |

## B. Details about self-attention mechanisms inside ABT-MPNN

**Table S3: Algorithm of Bond Attention**

| | |
|---|---|
| | **Initialization** |
| 1) | At iteration $t$, given a molecular graph $G$ with $N$ bonds, the input bond message matrix $H_b = [m_{b_1}^t, \dots, m_{b_i}^t, \dots, m_{b_{2N}}^t]$. $H_b \in \mathbb{R}^{2N \times d}$, where $d$ is the hidden dimension.<br>$Q_b = [q_{b_1}, \dots, q_{b_i}, \dots, q_{b_{2N}}] = H_b$<br>$K_b = [k_{b_1}, \dots, k_{b_i}, \dots, k_{b_{2N}}] = H_b$<br>$V_b = [v_{b_1}, \dots, v_{b_i}, \dots, v_{b_{2N}}] = H_b$ |
| | **Bond Attention** |
| 1) | for each bond message $b_i$: |
| 2) | $\alpha_{b_i} \leftarrow \frac{\exp(q_{b_i} w_q / \sqrt{d})}{\sum_{j=1}^{2N} \exp(q_{b_j} w_q / \sqrt{d})}$ |
| 3) | $q_b \leftarrow \sum_{i=1}^{2N} \alpha_{b_i} q_{b_i}$ |
| 4) | $p_{b_i} \leftarrow q_b * k_{b_i}$ |
| 5) | $\beta_{b_i} \leftarrow \frac{\exp(p_{b_i} w_k / \sqrt{d})}{\sum_{j=1}^{2N} \exp(p_{b_j} w_k / \sqrt{d})}$ |
| 6) | $k_b \leftarrow \sum_{i=1}^{2N} \beta_{b_i} q_{b_i}$ |
| 7) | $u_{b_i} \leftarrow k_b * v_{b_i}$ |
| 8) | $r_{b_i} \leftarrow u_{b_i} w_r + q_{b_i}$ |
| 9) | $O_b \leftarrow Layernorm([r_{b_1}, \dots, r_{b_{2N}}])$ |

**Table S4: Algorithm of Atom Attention**

| | |
|---|---|
| | **Initialization** |
| 1) | Given a molecular graph $G$ with $M$ atoms, the input atom hidden matrix $H_a = [m_{a_1}, \dots, m_{a_i}, \dots, m_{a_M}]$. $H_a \in \mathbb{R}^{M \times d}$, where $d$ is the hidden dimension.<br>$Q_a = K_a = V_a = H_a$ |
| 2) | If $head_1$ or $head_2$: $M_{graph} = \lambda \cdot M_{Adjacency} \in \mathbb{R}^{M \times M}$<br>If $head_3$ or $head_4$: $M_{graph} = \lambda \cdot M_{Distance} \in \mathbb{R}^{M \times M}$<br>If $head_5$ or $head_6$: $M_{graph} = \lambda \cdot M_{Coulomb} \in \mathbb{R}^{M \times M}$ |
| | **Atom Attention** |
| 1) | $A_a \leftarrow \frac{Q_a W_Q (K_a W_K)^T}{\sqrt{d}} + M_{graph}$ |
| 2) | $O_a \leftarrow Layernorm(Softmax(A_a) V_a W_V)$ |

## C. Details about the ablation study of ABT-MPNN

**Fig. S1 Comparison of ablation experiments using 5-fold cross-validation** (A) Performance evaluation of each fold for the classification task (ClinTox) measured with AUROC. Experiments settings: #1: baseline; #2: use bond attention (Transformer); #3: use bond attention (Fastformer); #4 use atom attention; #5 use atom attention with inter-atomic matrices #6 use bond attention (Fastformer) and atom attention; #7 use bond attention (Fastformer) and atom attention with inter-atomic matrices (B) Performance evaluation of each fold for the regression task (ESOL) measured by RMSE. The settings of each experiment in the regression task are identical to those in the classification one.

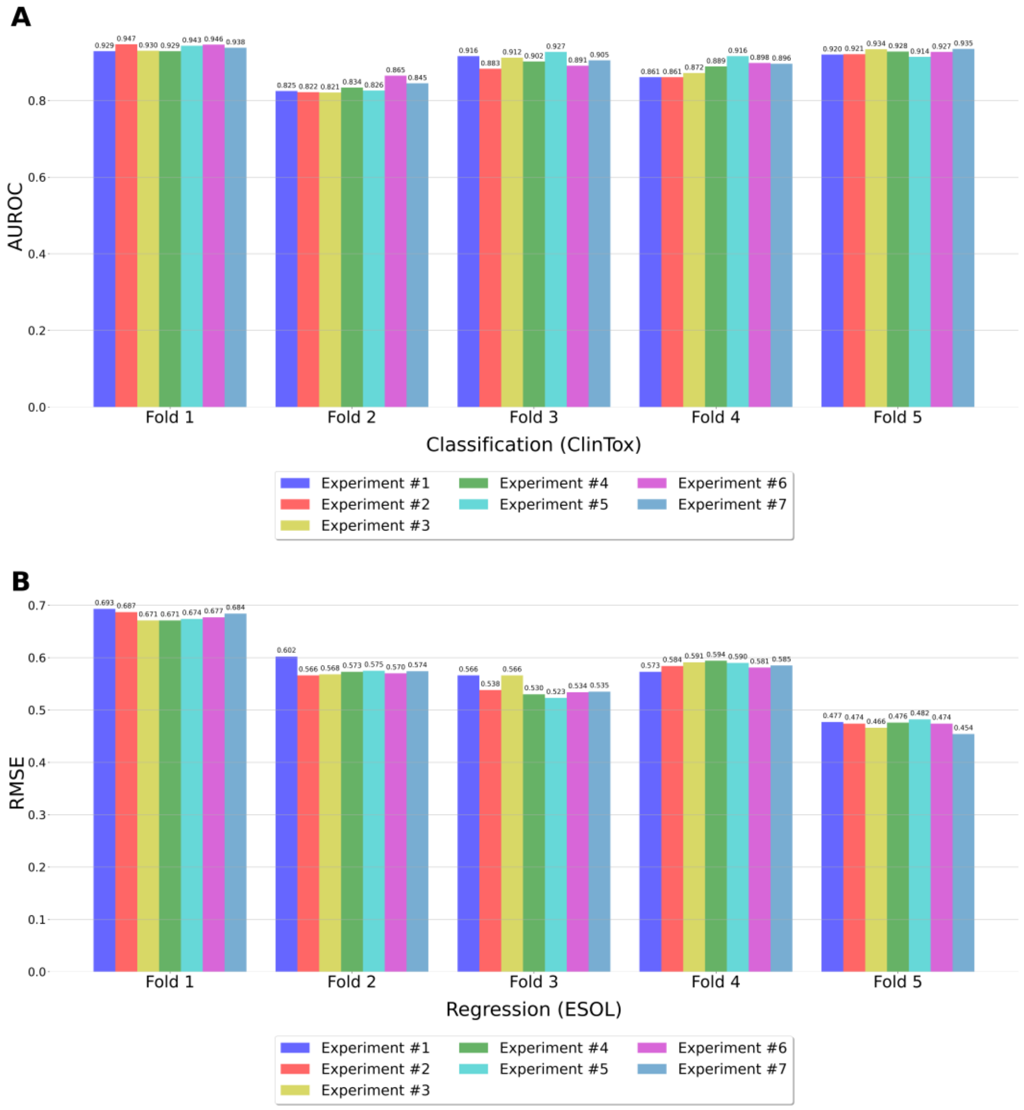

Supplement: Supplementary file 1 — Additional file 1: Table S1. Atom and bond features. Table S2. 200 Molecular descriptors generated by RDKit. Table S3. Algorithm of Bond Attention. Table S4. Algorithm of Atom Attention. Fig. S1. Comparison of ablation experiments using 5-fold cross-validation (A) Performance evaluation of each fold for the classification task (ClinTox) measured with AUROC. Experiments settings: #1: baseline; #2: use bond attention (Transformer); #3: use bond attention (Fastformer); #4 use atom attention; #5 use atom attention with inter-atomic matrices #6 use bond attention (Fastformer) and atom attention; #7 use bond attention (Fastformer) and atom attention with inter-atomic matrices (B) Performance evaluation of each fold for the regression task (ESOL) measured by RMSE. The settings of each experiment in the regression task are identical to those in the classification one. [file 13321_2023_698_MOESM1_ESM.pdf]
